# Performance on bedside tests of attention and organized thinking in patients with dementia free from delirium

**DOI:** 10.1017/S1041610218000522

**Published:** 2018-07-23

**Authors:** Letty Oudewortel, Karlijn J. Joling, Cees M. P. M. Hertogh, Viona J. M. Wijnen, Anne A. M. van der Brug, Willem A. van Gool

**Affiliations:** 1 Psychogeriatric Observation Unit, Institution for Mental Health Care ‘Dijk en Duin’, Parnassia Groep, Castricum, the Netherlands; 2 Department of General Practice & Elderly Care Medicine, Amsterdam Public Health Research Institute, VU University Medical Centre, Amsterdam, the Netherlands; 3 Nursing Home Facility and Elderly Care ViVa! Zorggroep, Heemskerk, the Netherlands; 4 Department of Neurology, Academic Medical Center, University of Amsterdam, Amsterdam, the Netherlands

**Keywords:** Delirium, dementia, severe dementia, delirium superimposed on dementia, attention, disorganized thinking, bedside test, test failure

## Abstract

**Objectives:**

Bedside tests of attention and organized thinking were performed in patients with cognitive impairment or dementia but without delirium, to provide estimates of false positive rates for detecting delirium superimposed on dementia (DSD).

**Design and Setting:**

This cross-sectional study was conducted in outpatients and institutionalized patients without delirium representing a wide spectrum of severity of cognitive impairments.

**Participants:**

Patients with dementia or a cognitive disorder according to DSM IV criteria, after exclusion of (suspected) delirium according to DSM IV criteria.

**Measurements:**

Tests for inattention and disorganized thinking from the CAM-ICU were assessed.

**Results:**

The sample included 163 patients (mean age 83 years (SD 6; 64% women)), with Alzheimer's disease as most prevalent (45%) diagnosis and a mean MMSE-score of 16.8 (SD 7.5). False positive rates of the test of attention varied from 0.04 in patients with normal to borderline cognitive function to 0.8 in those with severe dementia. The false positive rate of the test of disorganized thinking was zero in the normal to borderline group, increasing to 0.67 in patients with severe dementia. When combining test results false positive rates decreased to 0.03 in patients with MMSE scores above 9.

**Conclusion:**

Use of simple bedside tests of attention and organized thinking for the clinical diagnosis of DSD will result in high rates of false positive observations if used regardless of the severity of dementia. However, if test results are combined they may be useful to exclude DSD in patients with minimal to moderate degrees of dementia, but not in the severe group.

## Introduction

Delirium is a common and severe, neuropsychiatric syndrome in the elderly, characterized by fluctuating inattention, other cognitive deficits, altered arousal and hallucinations and/or delusions. Prevalence of delirium in older patients ranges from 14–56% (Fong *et al.*, 2009) and even up to 70–90% in patients with pre-existing dementia, depending on the severity of dementia (Voyer *et al.*, 2006). Older age and cognitive impairments are both important risk factors for delirium explaining the high prevalence of delirium superimposed on dementia (DSD) (Cole, 2004). DSD is associated with poor long-term clinical outcomes, such as accelerated decline in cognitive and physical functions, institutionalization and even death (Inouye *et al.*, 2006; Witlox *et al.*, 2010; Fick *et al.*, 2013; Davis *et al.*, 2017). The anxiety, hallucinations, and behavioral disturbances in DSD are associated with intense suffering in patients and increased burden in families and professional caregivers (Partridge *et al.*, 2013; Jans *et al.*, 2015). However, DSD often goes unrecognized by clinicians and nurses due to overlapping symptoms of delirium and dementia (Voyer *et al.*, 2007). Potentially, these diagnostic difficulties delay timely and appropriate counseling and treatment. Accurately delineating core features of delirium, such as newly impairments of attention with a fluctuating course as the most prominent features of delirium and decline of cognition and differentiating these from pre-existing cognitive deficits associated with underlying neurodegenerative or cerebrovascular disease, is obviously very difficult in daily medical practice (van Gool *et al.*, 2017)

Previous studies have reported the difficulties concerning the accurate diagnosis of DSD. In their review on the prevalence of DSD, Fick *et al.* (2002) concluded that the wide variation in prevalence likely reflects the use of numerous different screening tools to detect DSD. A large survey of DSD practice among international delirium specialists demonstrated that there is a lack of consensus concerning assessment and diagnosis of DSD. Richardson *et al.* (2016) and Morandi *et al.* (2017) concluded that the evidence base for tools to detect DSD is limited, and constitutes an emerging challenge. In the absence of specific DSD tools, recommendations were made to focus on attention (Fick *et al.*, 2013; Tieges *et al.*, 2014; Richardson *et al.*, 2017), and disorganized thinking (Voyer *et al.*, 2006; Meagher *et al.*, 2012; Fick *et al.*, 2013), in order to differentiate coexisting delirium from isolated premorbid dementia.

Several studies evaluated the value of bedside tests covering attention and organization of thinking like for example the confusion assessment method for the intensive care unit (CAM-ICU) (Ely *et al.*, 2001) in differentiating symptoms of DSD from pre-existing cognitive impairment or dementia (Meagher *et al.*, 2010; Morandi *et al.*, 2012; Leonard *et al.*, 2016; Richardson *et al.*, 2017). However, most of these studies have been performed in hospitalized patients, with a retrospectively determined dementia diagnosis and patients with a pre-existing severe dementia were under-represented in these studies (Morandi *et al.*, 2012). Therefore, essential information is not available that may serve to gauge the potential value of specific bedside tests advocated for the detection of DSD. Information on the capability of passing tests of attention and organization of thinking like used in the cognitively impaired subjects without delirium is essential for assessing their potential specificity in reliably establishing a diagnosis of DSD. To this end, this study aims to provide estimates of potential false positive rates for detecting DSD by evaluating test performance on simple and widely used clinical executive tests of attention and organized thinking both in outpatients and institutionalized patients without delirium, across a wide spectrum of severity of cognitive impairments.

## Methods

### Study sample and design

In this descriptive cross-sectional study, attention and organization of thinking were tested in patients with cognitive impairment and/or dementia, but free from delirium, in order to examine the false positive (and true negative) rates if these tests would be used to detect DSD. Patients were recruited between January 2015 and April 2016.

### Study participants and settings

Participants were recruited from two settings: a geriatric outpatient service (GOS) for cognitive evaluation and a long-term care facility (LTCF) for people with dementia, both in the Netherlands. Patients were eligible for the study if dementia or a cognitive disorder was diagnosed and classified by an elderly care physician (Koopmans *et al.*, 2010) according to Diagnostic and Statistical Manual of Mental Disorders IV (DSM IV) criteria (AmericanPsychiatricAssociation, 1994). Exclusion criteria are as follows: (1) current delirium as assessed by an elderly care physician or trained psychologist using the DSM IV criteria for delirium, or (2) suspected delirium in the weeks preceding assessment, according to information obtained by an interview with the primary caregiver (in the GOS setting) or nurses (in the LTCF), or (3) any condition precluding proper test interpretation like e.g. concomitant severe psychiatric disorder or language barrier. Informed consent was obtained from patients, in case of decisional incapacity, consent was derived from the legal representatives. The ethics committee of the VU University Medical Center reviewed the study.

### Procedures

All patients referred to the GOS with a history of cognitive impairment or (suspected) dementia were examined by an elderly care physician. In addition to GOS standard diagnostic assessments such as the Mini-Mental State Examination (MMSE) (Folstein *et al.*, 1975) patients were invited to participate in the assessments for the current study. Caregivers were interviewed by a psychiatric nurse to obtain information concerning the cognitive impairment, its course and on comorbid conditions. In addition, to determine the severity of dementia people were assessed with the cognitive performance scale (CPS) (Morris *et al.*, 1994) and in order to rule out a current or recent episode of delirium, specific questions were asked about delirium features, like acute change or fluctuations in cognitive or psychological performance, for the past few weeks. The results of all assessments were discussed in a multidisciplinary team with participation of an elderly care physician, a neuropsychologist, psychiatrist and psychiatric nurse. Diagnoses of dementia or cognitive disorders were classified according DSM IV criteria.

For patients living in the LTCF, an elderly care physician or psychologist did the assessments by performing the MMSE and ruling out or making a delirium diagnosis according DSM IV criteria. Nurses of the department were asked to fill in the CPS and they were interviewed to determine possible delirium features over the preceding few weeks. The dementia diagnoses were obtained from the medical records.

### Measurements

The tests under investigation probing inattention and disorganized thinking were both taken from the CAM-ICU, which has been proposed as a test to detect DSD (Morandi *et al.*, 2012).

### Attention

Attention was tested by asking the patient to hold the examiner's hand, saying: “I am going to read you a series of 10 letters. Whenever you hear the letter ‘A’ indicate so by squeezing my hand.” Followed by listing “C-A-S-A-B-L-A-N-C-A,” in a normal tone, each letter 2–3 sec apart. Errors were counted when patients failed to squeeze on the letter “A” and when patients squeezed on any other letter. If a patient made more than two mistakes the test was scored as abnormal.

### Organization or coherence of thinking

Organization or coherence of thinking was tested by first asking the patient a “yes” or “no” answer to the following four questions: “Will a stone float on water?,” “Are there fish in the sea?,” “Does one pound weigh more than two pounds?,” “Can you use a hammer to pound a nail?” More than one error on the combined four questions was interpreted as evidence of disorganized thinking. If a patient responded correctly to three or all four questions, the patient was asked to fulfill the following commands: first “Hold up this many fingers” when the examiner held up two fingers. Next the patient was asked: “Now do the same with your other hand”, without giving an example. Both commands must be responded correctly to pass the test.

### Severity of cognitive impairment

The MMSE was assessed and adapted Perneckzy criteria (Perneczky *et al.*, 2006) were applied to categorize scores into four categories: no or questionable dementia (score of 30–25), mild (24–21), moderate (20–10), or severe dementia (9–0).

Since it was anticipated that it might be difficult to appropriately assess the MMSE in some participants from the LTCF, we also applied the CPS for grading the severity of dementia (Morris *et al.*, 1994). The CPS is a hetero-anamnestic list validated for LTCF settings. CPS scores correspond closely to those generated by the MMSE (Hartmaier *et al.*, 1995). The CPS can be classified into seven cognitive performance categories. In line with the previous research, these categories were further collapsed into four levels of impairment for this study because of the small number of participants in some classes: (1) normal/questionable (combining “intact”; and “borderline intact” on the CPS), (2) mild, (3) moderate (both according to the existing CPS categories), (4) severe (collapsing “moderate or severe impairment,” “severe impairment,” and “very severe impairment” of the original scale) (Teno *et al.*, 2010).

### Statistical analyses

We investigated baseline similarity in the characteristics of the persons who were excluded (*n* = 44) and the study sample (*n* = 163) to examine potential selection bias by using *χ*^2^ tests for categorical variables, and independent *t*-tests for continuous variables, or Mann–Whitney tests if continuous baseline variables were skewed. Descriptive statistics were used to describe socio-demographical and clinical characteristics of the study sample. To estimate the potential value of the tests for attention and organized thinking for detecting delirium superimposed dementia we calculated the rates of false positives and true negatives for the four MMSE and CPS categories. The specificity was calculated by 1-specificity = FPR. The association between the severity of cognitive impairment (as measured with the MMSE and CPS) and false positive rates of the two detecting tests were analyzed by correlation testing (Spearman's *ρ*). SPSS (IBM version 22) was used for all statistical analyses.

## Results

**Figure 1.**
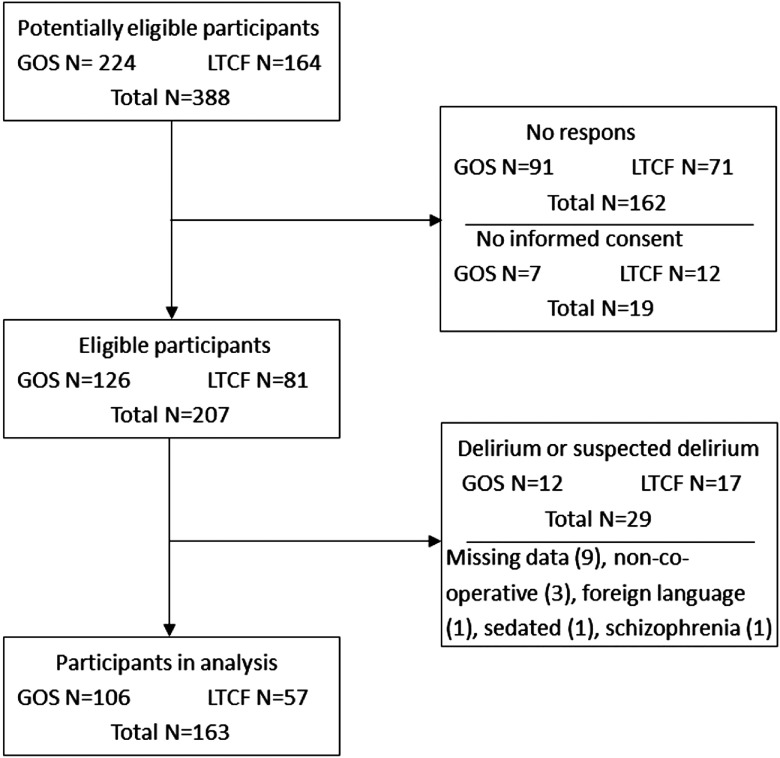
Flow chart.

A total of 207 (53%) participants from the 388 potentially eligible subjects were assessed (see Figure 1). Delirium was diagnosed or suspected in 29 patients, and these persons were thus excluded for analysis. Another 15 patients were excluded because of various reasons specified in the flow diagram (Figure 1). As a result, 163 patients were included in the analysis. Persons who were excluded (*n* = 44) had significantly more severe cognitive impairment, both on the MMSE (mean MMSE score 16.8 vs. 10.2, 95% CI 3.45–9.80, p < 0.001) and CPS (mean CPS score 2.4 vs. 3.6, 95% CI −1.81 to −0.68, p < 0.001). There were no significant differences between both groups with regard to age, gender, and recruitment setting.

### Participants characteristics

Table 1 shows the socio-demographic characteristics, the diagnoses, and the distribution among severity categories of the study sample in total and divided by origin. The study participants showed a large variation of ages (range 65–101 years, mean 83 years, standard deviation (SD) ±6 years) and dementia stage (Table 1). Alzheimer's disease was the most frequent dementia diagnosis (45%), followed by dementia not otherwise specified (NOS) (17%), cognitive disorder NOS (13%) and vascular dementia (10%).

**Table 1.** Demographic and clinical characteristics of study participants

characteristic	total sample (*n* = 163)	geriatric outpatients (*n* = 106)	LTCF residents (*n* = 57)
Age, years (Mean ±SD)	83.5 ± 6	82.9 ± 6	84.6 ± 7
Gender, *N* (%) females	105 (64)	66 (62)	39 (68)
Diagnoses, *N* (%)			
Alzheimer disease	73 (45)	41 (39)	32 (56)
Cognitive disorder NOS	21 (13)	20 (19)	1 (2)
Dementia NOS	28 (17)	17 (16)	11 (19)
Frontotemporal dementia	1 (1)	0 (0)	1 (2)
Mild cognitive impairment	5 (3)	5 (5)	0 (0)
Mixed dementia	10 (6)	8 (7)	2 (4)
Parkinson's dementia	3 (2)	2 (2)	1 (2)
Vascular dementia	17 (10)	8 (8)	9 (16)
Vascular cognitive impairment	3 (2)	3 (3)	0 (0)
MMSE mean (±SD)	16.8 (±7.5)	20.6 (±4.6)	10.0 (±7.0)
MMSE level, *N* (%)			
30–25 Normal/questionable	24 (15)	23 (22)	1 (2)
24–21 Mild	40 (25)	35 (33)	5 (9)
20–10 Moderate	66 (41)	43 (41)	23 (40)
9–0 Severe	30 (18)	2 (2)	28 (49)
CPS category, *N* (%)			
0–1 Normal/borderline	30 (18)	24 (23)	6 (10)
2 Mild	75 (46)	57 (54)	18 (32)
3 Moderate	27 (17)	9 (9)	18 (32)
4,5,6 (Very) severe	25 (15)	10 (9)	15 (26)

### Attention test

Fail rate of the attention test in the group for whom the CPS was available (*n* = 157) was in total 0.31, equivalent to specificity of 0.69. Depending on the severity of the cognitive impairment the fail rates ranged from 0.1 (0.90) in patients with no to borderline dementia to 0.72 (0.28) in those with (very) severe dementia (*ρ* = Spearman correlation 0.48; p < 0.001).

Classification according to severity categories based on the MMSE score yielded fail rates varying from 0.04 (0.96) in the normal/questionable group to 0.8 (0.2) in subjects in the severe group (*ρ* = 0.53; p < 0.001). For the total group (*n* = 160) the false positive rate was 0.29, equivalent to specificity of 0.69 (Table 2).

**Table 2.** Fail rates of tests of attention or organized thinking in subjects with dementia, without delirium

level of impairment	*N*	fail rate test of attention (95% CI)	spearman correlation	fail rate test of organized thinking (95% CI)	spearman correlation
CPS staging (rate, CI)					
Normal/borderline	30	0.10 (0.03–0.26)		0.03 (0.01–0.17)	
Mild	75	0.16 (0.09–0.26)		0.09 (0.05–0.18)	
Moderate	27	0.56 (0.37–0.72)		0.56 (0.37–0.72)	
(Very) severe	25	0.72 (0.52–0.86)		0.52 (0.34–70)	
All	157**	0.31 (0.24–0.38)	0.478*	0.23 (0.17–0.30)	0.471*
MMSE staging (rate, CI)					
30–25 Normal/questionable	24	0.04 (0.01–0.20)		0.00 (0.00–0.13)	
24–21 Mild	40	0.08 (0.02–0.20)		0.08 (0.03–0.20)	
20–10 Moderate	66	0.27 (0.18–0.39)		0.17 (0.10–0.27)	
9–0 Severe	30	0.80 (0.63–0.90)		0.67 (0.49–0.81)	
All	160**	0.29 (0.22–0.36)	0.531*	0.21 (0.16–0.28)	0.480*

*p < 0.001; **6 out of 163 CPS cores were missing and 3 out of 163 MMSE scores were missing. CI: confidence interval.

### Organization of thinking test

Evidence for disorganized thinking was present in 0.23, equivalent to specificity of 0.77 in the total group assessed with the CPS, with 0.03 (0.97) of patients with impairments falling in the lowest CPS category and this increased to 0.52 (0.48) in the most severe category of severity (*ρ* = 0.47; p < 0.001). In the total group with a MMSE score 0.21, fail rates were found equivalent to specificity of 0.79., In the normal/questionable group, no subjects failed the test for organized thinking, but the fail rate increased from 0.08 (0.92) in the mild group via 0.17 (0.83) in the moderate group to 0.67 (0.33) in the severe group (*ρ* = 0.48; p < 0.001) (Table 2).

### Combined test results

**Figure 2.**
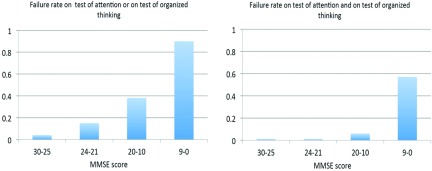
Failure rates on combined test.

Rates for failing either the test for attention or the test for organized thinking are depicted in Figure 2 per MMSE severity category, mounting up to 0.9, equivalent to specificity of 0.1 for patients with the lowest MMSE scores. Failing both tests occurred in 0.57, equivalent to specificity of 0.43, of these patients, but only 4 of the 130 participants with MMSE scores above 9 failed both tests (Figure 2 right panel).

## Discussion

The objective of this study was to examine the capability of patients without delirium within a wide range of cognitive impairment to correctly perform two simple bedside tests for attention and organization of thinking. Both tests are widely used to capture episodes of delirium as part of the CAM-ICU. If these patients fail to successfully complete these tests, this provides an estimate of potential false positive rates for detecting DSD. In this way, their potential value in reliably establishing a diagnosis of DSD could be explored.

As far as we know, this study is the first to address this question specifically in a substantial group of patients with a DSM IV diagnosis of cognitive impairment from minimal to severe dementia in whom delirium was carefully excluded. Previous studies reported positive tests results for detecting DSD in patients without specifying the severity of dementia (Bellelli *et al.*, 2014; Adamis *et al.*, 2016; O'Regan *et al.*, 2016) or in groups of patients with (moderate to severe) dementia that were relative small (Leonard *et al.*, 2016; Voyer *et al.*, 2016; Richardson *et al.*, 2017).

We found substantial false positive rates on the tests of attention and organized thinking, which were both positively associated with increasing severity of dementia. This can be explained by the fact that attention is compromised in the moderate and severe stages of dementia (Perry *et al.*, 2000; Kolanowski *et al.*, 2012). In addition, the capability to organized thinking also depends on the degree of global cognitive functioning (Bhat and Rockwood, 2007). As the study participants represent a wide spectrum of ages and clinical characteristics, these findings indicate that the tests under study result in high false positive rates if used without taking into account the severity of cognitive impairment to detect DSD. Our findings are in line with study results showing that a letter recognition test distinguished patients with delirium from those with dementia but also resulted in high false positive rates (Leonard *et al.*, 2016). By evaluation of five attention measurements, Adamis *et al.* (2016) found 40–50% positive test results for inattention in patients with dementia who were free from delirium symptoms. Consistent with the present results, Voyer *et al.* (2016) found a substantial decrease in specificity on the month of the year backwards MOTYB task in patients with cognitive impairment.

However, our findings indicate that false positive rates may be reduced substantially when the results of both tests are interpreted in combination rather than as isolated findings in patients with minimal, mild, and moderate dementia. In patients without delirium but with MMSE scores below 10, both tests appear not useful for excluding delirium because false positive rates between 0.67 and 0.80 can be expected.

In recent research, Richardson *et al.* (2017) demonstrated that the combination of a letter recognition attention test (similar to the test used in our study) with The Observational Scale of Level of Arousal (OSLA) (Tieges *et al.*, 2013) performed better than the two test individually. The observational nature of the OSLA requires only minimal cooperation of subjects and level of arousal is closely associated with attentional deficits in delirium (Boustani *et al.*, 2014). It is promising to further evaluate combinations of letter recognition, disorganization of thinking, and the OSLA in the population of patients with severe cognitive impairments in order to lower the rate of false positives.

Furthermore, it is important to emphasize that the test evaluated here are only parts of the diagnostic algorithm for delirium. Positive test results can contribute to the diagnosis especially in DSD; however, diagnosing DSD always needs more extensive clinical assessment.

In our study, we had to exclude 20% of subjects (17 out of 81 patients) from the LTCF-population because of (suspected) delirium. It is important to note that these delirium cases were unrecognized by nurses or physicians since we screened the population in order to exclude the delirium diagnosis for the study. Better recognition by healthcare professionals is needed for timely and appropriate counseling and treatment of the syndrome.

The study knows some limitations The assessments to exclude delirium proceeded through clinical investigation applying DSM IV criteria and interviews with nurses (in LTCF) or caregivers (in subjects visiting the GOS). These interviews focused on possible signs or symptoms of delirium over the preceding few weeks. Especially in the LTCF setting, the outcome of the interview may be limited in reliability due to shift work of the nurses. However, combining this information with the clinical examination provided satisfactory outcome as exemplified by exclusion of 20% of the LTCF subjects because of suspected current or recent delirium.

Although the heterogeneity of our study population could also be considered a study limitation we believe that these variation in ages, diagnoses, and severity of impairments adds to the external validity of our findings for everyday clinical practice both in GOSs as well as in long term care facilities.

Grading the severity of dementia especially in the LTCF is difficult. Assessing executive test can be followed by noncompliance because of understanding problems or resistance. We chose two complementary approaches by using the MMSE, an executive test restricting the grading to cognitive impairments per se, and the CPS an observational test that can be completed regardless of the severity of cognitive impairments. Regardless of the measurement instrument, we found, increasing rates of test failure on both tests with increasing severity of cognitive impairment.

A strength of our study is that we were able to establish accurate dementia diagnoses and we could classify subjects according to the degree of severity of dementia, rather than retrospective classifications as in some previous studies (Bellelli *et al.*, 2014; De *et al.*, 2016; Morandi *et al.*, 2016; O'Regan *et al.*, 2016). By excluding delirium or suspected delirium the study provides information about potential false positive rates of well recommended simple tests for delirium in a population representing a wide spectrum of ages, diagnoses, and severity of cognitive impairments. Because we performed the test across the complete range of cognitive impairments we are now able to characterize potential false positive rates (for diagnosing DSD) in subjects with severe dementia a group that has been under-researched so far.

We conclude that use of simple bedside tests of attention and organized thinking for detecting DSD will lead to disproportionally high rates of false positive observations if used in isolation in patients across a wide spectrum of degrees of dementia. However, if test results are evaluated in combination they may serve to exclude DSD with confidence in patients with minimal, mild, and moderate degrees of dementia. The simple bedside tests under investigation are not suited to exclude delirium in subjects with severe degrees of dementia. In the latter populations, it may be worthwhile to combine results of bedside examinations with observation of levels of arousal (Tieges *et al.*, 2013).

## Conflict of interest

None.

## Description of authors’ roles

Study conception and design was done by LO, WAvG, KJ, and CH. Acquisition of data was done by LO and AB. Data analysis was done by LO, KJ, and WAvG. Interpretation of results, critical revision, and final approval of the manuscript was done by all the authors.

## References

[ref001] Adamis D. et al (2016). Evaluating attention in delirium: a comparison of bedside tests of attention. Geriatrics and Gerontology International, 16, 1028–1035. 26419620 10.1111/ggi.12592

[ref002] American Psychiatric Association (1994). Diagnostic and Statistical Manual of Mental Disorders, Fourth Edition (DSM-IV). Washington, DC: American Psychiatric Publishing, Inc.

[ref003] Bellelli G. et al (2014). Validation of the 4AT, a new instrument for rapid delirium screening: a study in 234 hospitalised older people. Age Ageing, 43, 496–502. 24590568 10.1093/ageing/afu021 PMC4066613

[ref004] Bhat R. and Rockwood K. (2007). Delirium as a disorder of consciousness. Journal of Neurology, Neurosurgery, and Psychiatry, 78, 1167–1170. 10.1136/jnnp.2007.115998 PMC2117593 17488786

[ref005] Boustani M. et al (2014). The DSM-5 criteria, level of arousal and delirium diagnosis: inclusiveness is safer. BMC Medicine, 12, 141. 25300023 10.1186/s12916-014-0141-2 PMC4177077

[ref006] Cole M. G. (2004). Delirium in elderly patients. American Journal of Geriatric Psychiatry, 12, 7–21. 14729554

[ref007] Davis D. H. et al (2017). Association of delirium with cognitive decline in late life: a neuropathologic study of 3 population-based cohort studies. JAMA Psychiatry, 74, 244–251. 28114436 10.1001/jamapsychiatry.2016.3423 PMC6037291

[ref008] De J., Wand A. P., Smerdely P. I. and Hunt G. E. (2016). Validating the 4A's test in screening for delirium in a culturally diverse geriatric inpatient population. International Journal of Geriatric Psychiatry, 32, 1322–1329. 27766672 10.1002/gps.4615

[ref009] Ely E. W. et al (2001). Delirium in mechanically ventilated patients: validity and reliability of the confusion assessment method for the intensive care unit (CAM-ICU). JAMA, 286, 2703–2710. 11730446 10.1001/jama.286.21.2703

[ref010] Fick D. M., Agostini J. V. and Inouye S. K. (2002). Delirium superimposed on dementia: a systematic review. Journal of the American Geriatrics Society, 50, 1723–1732. 12366629 10.1046/j.1532-5415.2002.50468.x

[ref011] Fick D. M., Steis M. R., Waller J. L. and Inouye S. K. (2013). Delirium superimposed on dementia is associated with prolonged length of stay and poor outcomes in hospitalized older adults. Journal of Hospital Medicine, 8, 500–505. 23955965 10.1002/jhm.2077 PMC3928030

[ref012] Folstein M. F., Folstein S. E. and McHugh P. R. (1975). “Mini-mental state”. A practical method for grading the cognitive state of patients for the clinician. Journal of Psychiatric Research, 12, 189–198. 1202204 10.1016/0022-3956(75)90026-6

[ref013] Fong T. G., Tulebaev S. R. and Inouye S. K. (2009). Delirium in elderly adults: diagnosis, prevention and treatment. Nature Reviews Neurology, 5, 210–220. 19347026 10.1038/nrneurol.2009.24 PMC3065676

[ref014] Hartmaier S. L., Sloane P. D., Guess H. A., Koch G. G., Mitchell C. M. and Phillips C. D. (1995). Validation of the minimum data set cognitive performance scale: agreement with the mini-mental state examination. Journals of Gerontology. Series A, Biological Sciences and Medical Sciences, 50, M128–133. 10.1093/gerona/50a.2.m128 7874589

[ref015] Inouye S. K., Zhang Y., Han L., Leo-Summers L., Jones R. and Marcantonio E. (2006). Recoverable cognitive dysfunction at hospital admission in older persons during acute illness. Journal of General Internal Medicine, 21, 1276–1281. 16965558 10.1111/j.1525-1497.2006.00613.x PMC1924736

[ref016] Jans I. S., Oudewortel L., Brandt P. M. and van Gool W. A. (2015). Severe, persistent and fatal delirium in psychogeriatric patients admitted to a psychiatric hospital. Dementia and Geriatric Cognitive Disorders Extra, 5, 253–264. 26195981 10.1159/000381847 PMC4483487

[ref017] Kolanowski A. M., Fick D. M., Yevchak A. M., Hill N. L., Mulhall P. M. and McDowell J. A. (2012). Pay attention! The critical importance of assessing attention in older adults with dementia. Journal of Gerontological Nursing, 38, 23–27. 23066682 10.3928/00989134-20121003-05 PMC3571625

[ref018] Koopmans R. T., Lavrijsen J. C., Hoek J. F., Went P. B. and Schols J. M. (2010). Dutch elderly care physician: a new generation of nursing home physician specialists. International Journal of Geriatric Psychiatry, 58, 1807–1809. 10.1111/j.1532-5415.2010.03043.x 20863347

[ref019] Leonard M. et al (2016). Attention, vigilance and visuospatial function in hospitalized elderly medical patients: relationship to neurocognitive diagnosis. Journal of Psychosomatic Research, 90, 84–90. 27772564 10.1016/j.jpsychores.2016.09.011

[ref020] Meagher D. J., Leonard M., Donnelly S., Conroy M., Saunders J. and Trzepacz P. T. (2010). A comparison of neuropsychiatric and cognitive profiles in delirium, dementia, comorbid delirium-dementia and cognitively intact controls. Journal of Neurology, Neurosurgery, and Psychiatry, 81, 876–881. 10.1136/jnnp.2009.200956 20587481

[ref021] Meagher D., Adamis D., Trzepacz P. and Leonard M. (2012). Features of subsyndromal and persistent delirium. British Journal of Psychiatry, 200, 37–44. 22075650 10.1192/bjp.bp.111.095273

[ref022] Morandi A. et al (2012). Tools to detect delirium superimposed on dementia: a systematic review. Journal of the American Geriatrics Society, 60, 2005–2013. 23039270 10.1111/j.1532-5415.2012.04199.x PMC3498536

[ref023] Morandi A. et al (2016). Detecting delirium superimposed on dementia: evaluation of the diagnostic performance of the richmond agitation and sedation scale. Journal of the American Medical Directors Association, 17, 828–833. 27346621 10.1016/j.jamda.2016.05.010 PMC5257263

[ref024] Morandi A. et al (2017). The diagnosis of delirium superimposed on dementia: an emerging challenge. Journal of the American Medical Directors Association, 18, 12–18. 27650668 10.1016/j.jamda.2016.07.014 PMC5373084

[ref025] Morris J. N. et al (1994). MDS cognitive performance scale. Journal of Gerontology, 49, M174–182. 8014392 10.1093/geronj/49.4.m174

[ref026] O'Regan N. A. et al (2016). Five short screening tests in the detection of prevalent delirium: diagnostic accuracy and performance in different neurocognitive subgroups. International Journal of Geriatric Psychiatry, 32, 1440–1449. 27917538 10.1002/gps.4633

[ref027] Partridge J. S., Martin F. C., Harari D. and Dhesi J. K. (2013). The delirium experience: what is the effect on patients, relatives and staff and what can be done to modify this? International Journal of Geriatric Psychiatry, 28, 804–812. 23112139 10.1002/gps.3900

[ref028] Perneczky R., Wagenpfeil S., Komossa K., Grimmer T., Diehl J. and Kurz A. (2006). Mapping scores onto stages: mini-mental state examination and clinical dementia rating. American Journal of Geriatric Psychiatry, 14, 139–144. 16473978 10.1097/01.JGP.0000192478.82189.a8

[ref029] Perry R. J., Watson P. and Hodges J. R. (2000). The nature and staging of attention dysfunction in early (minimal and mild) Alzheimer's disease: relationship to episodic and semantic memory impairment. Neuropsychologia, 38, 252–271. 10678692 10.1016/s0028-3932(99)00079-2

[ref030] Richardson S. J. et al (2017). Detecting delirium superimposed on dementia: diagnostic accuracy of a simple combined arousal and attention testing procedure. International Psychogeriatrics, 29, 1585–1593. 28560945 10.1017/S1041610217000916

[ref031] Richardson S. et al (2016). Delirium superimposed on dementia: a survey of delirium specialists shows a lack of consensus in clinical practice and research studies. International Psychogeriatrics, 28, 853–861. 26692021 10.1017/S1041610215002288

[ref032] Teno J. M. et al (2010). Hospital characteristics associated with feeding tube placement in nursing home residents with advanced cognitive impairment. JAMA, 303, 544–550. 20145231 10.1001/jama.2010.79 PMC2847277

[ref033] Tieges Z., Brown L. J. and MacLullich A. M. (2014). Objective assessment of attention in delirium: a narrative review. International Journal of Geriatric Psychiatry, 29, 1185–1197. 24760756 10.1002/gps.4131

[ref034] Tieges Z., McGrath A., Hall R. J. and Maclullich A. M. (2013). Abnormal level of arousal as a predictor of delirium and inattention: an exploratory study. American Journal of Geriatric Psychiatry, 21, 1244–1253. 24080383 10.1016/j.jagp.2013.05.003

[ref035] van Gool W. A., Oudewortel L., and Hertogh C. P. M. (2017). Delirium, dementia and “. . . I knew there was but one way” delirium superimposed on dementia a conceptual approach. Gerontology and Geriatric Research, 1, 112.

[ref036] Voyer P. et al (2016). Assessment of inattention in the context of delirium screening: one size does not fit all!. International Psychogeriatric, 28, 1293–1301. 10.1017/S1041610216000533 27004924

[ref037] Voyer P., Cole M. G., McCusker J. and Belzile E. (2006). Prevalence and symptoms of delirium superimposed on dementia. Clinical Nursing Research, 15, 46–66. 16410622 10.1177/1054773805282299

[ref038] Voyer P., Doucet L., Danjou C., Cyr N. and Benounissa Z. (2007). [Detection of delirium by nurses]. Perspective Infirmiere, 5, 12–20. 18236878

[ref039] Witlox J., Eurelings L. S. M., de Jonghe J. F. M., Kalisvaart K. J., Eikelenboom P. and van Gool W. A. (2010). Delirium in elderly patients and the risk of postdischarge mortality, institutionalization, and dementia: a meta-analysis. JAMA, 304, 443–451. 20664045 10.1001/jama.2010.1013

